# Visual short-term memory is modulated by 3D depth in stereopsis

**DOI:** 10.3758/s13414-025-03052-3

**Published:** 2025-08-08

**Authors:** Hang Liu, Bruno Laeng, Nikolai Olavi Czajkowski

**Affiliations:** 1 Department of Psychology, University of Oslo, Forskningsveien 3A, 0373, Oslo, Norway; 2 RITMO Centre for Interdisciplinary Studies in Rhythm, Time and Motion, Oslo, Norway; 3 Department of Mental Health, Norwegian Institute of Public Health, Oslo, Norway; 4 Department of Psychology, PROMENTA Research Center, University of Oslo, Oslo, Norway

**Keywords:** Visual short-term memory, Stereopsis, 3D depth, Virtual reality

## Abstract

Visual short-term memory (VSTM) is a memory mechanism temporarily holding visual information for later use. In the real world, objects are located in spatial depth and such depth information may be processed when objects are remembered. The present study investigated how VSTM is modulated by three-dimensional (3D) depth. Previous work used the color of objects to measure memory and provided mixed results, and the impact of separability in depth on memory is unclear. In Experiment 1a, we employed color feature and examined the effect of 3D depth across Stereo (stereoscopic vs. monoscopic) and Plane (one plane vs. multiple planes). VSTM was not found to be influenced by depth information. In Experiments 1b and 1c, the color was respectively replaced with two distinct spatial features, orientation and direction-of-rotation. We found similar results that the performance on VSTM was superior in the stereoscopic multi-plane condition compared to the monoscopic multi-plane condition or stereoscopic single-plane condition. These findings confirm, also via Bayesian statistical analyses, that the VSTM can benefit from 3D depth information in stereopsis, while the benefit is severely limited when the task is non-spatial.

## Introduction

Remembering what we have just seen, but which is no longer visible, plays an essential role in visual cognition. Indeed, a specific visual short-term memory (VSTM) has been posited to maintain sensory information which is no longer directly available, at least for a very short time (Phillips & Baddeley, 1971). In order to measure VSTM ability, participants are often shown a memory array (e.g., squares, letters, or numerals) and instructed to remember a specific feature (e.g., name, color, or orientation) (e.g.Shin et al., 2017; Simons & Levin, 1997; Zhang & Luck, 2008). One of the consistent findings from this research is that the capacity of VSTM seems very limited, as many studies indicate that on average only three to five items can be actively held in mind (Alvarez & Cavanagh, 2004; Awh et al., 2007; Luck & Vogel, 1997). VSTM capacity is influenced by factors such as selective attention, fluid intelligence, and individual strategies (Downing, 2000; Fukuda et al., 2010; Vogel et al., 2005) as well as parameters of visual stimuli like the size of memory array and duration of stimulus presentation (Ma et al., 2014; Panichello et al., 2019; Shin et al., 2017).

In most VSTM studies, visual stimuli or objects are presented figuratively on a two-dimensional (2D) or “flat” screen, although in the real world, objects to be remembered are solid and positioned in physical three-dimensional (3D) space. Ecological human vision integrates the 2D, monocular, retinal images in a 3D, binocular, perception of the visual field as a “fused” representation of both images from both eyes. Binocular vision delivers a vivid perception of the three-dimensionality of objects, termed stereopsis, built upon the slight disparities of our eyes’ visual fields. One key question is the function of stereopsis in perception but also in memory. There is evidence demonstrating that achieving stereoscopic vision may facilitate the identification of natural objects as well as faces at the individual level (Caziot & Backus, 2015; Chelnokova & Laeng, 2011; Liu et al., 2020; Lim Lee & Saunders, 2011). Stereopsis also improves the performance in visual search tasks (Dent et al., 2012; Finlayson & Grove, 2015; Finlayson et al., 2013; Godwin et al., 2017). It is therefore reasonable to think that stereoscopic information may also be stored in VSTM and play a role in its function. Only a few recent studies have addressed this question by employing a stereoscope or virtual reality (VR) head-mounted displays (HMDs) to present visual stimuli in stereopsis (Chunharas et al., 2019; Qian & Zhang, 2019; Qian et al., 2018; Reeves & Lei, 2014, 2017; Sarno et al., 2019; Xu & Nakayama, 2007). However, none of these directly contrasted a stereoscopic condition with a monoscopic condition (i.e., where there is no disparity between visual information from the two eyes). Yet, this seems an essential comparison in order to test the role of stereoscopic vision in perception and memory. Thus, one of the goals of the current set of experiments was to address whether VSTM benefits from binocular vision or not compared to monocular information.

Previous studies that displayed stimuli in stereopsis provided mixed results for the effect of 3D depth information on the capacity of VSTM. Xu and Nakayama (2007) found that the number of items retained in VSTM increased when memory items were located in two depth planes compared to the condition that all items were on a same plane. Sarno, Lewis, and Neider’s study (2019) presented visual stimuli in a single-plane condition or multiple-plane condition and found that the memory for a large set of objects was facilitated by distinct depth planes. A recent study using a virtual reality (VR) display to present memory items in stereopsis showed that separating the items in depth enhances performance on VSTM (Chunharas et al., 2019), possibly by reducing interference between them. However, several studies have also supported the opposite conclusion that VSTM may work independently of depth information (Qian & Zhang, 2019; Reeves & Lei, 2014). Moreover, the results of some studies indicate that such 3D depth information may only be helpful in specific conditions. In Qian and colleagues’ study (2017), participants’ performance on the VSTM task was only better when remembering items in a closer plane compared to items in a more distant plane. Another study showed that the effect of 3D depth on VSTM was largely influenced by the saturation and brightness of visual stimuli (Qian et al., 2018). Taken together, it is still unclear how the capacity of VSTM is modulated by the presence of 3D depth.

Reviewing the previous VSTM literature, it appears that most experiments have utilized the change-detection paradigm, where the task for the participants is to determine whether the color of objects have changed or remain the same (Chunharas et al., 2019; Qian et al., 2017, 2018; Sarno et al., 2019; Xu & Nakayama, 2007). However, color may be a poor feature for shedding light on the effects of 3D depth on VSTM, since the color feature of an object is not a spatial feature. Thus, in order to explore the relationship between depth and VSTM, we employed, in addition to color, two other clearly spatial features, the object’s orientation and its rotational axis. In the current study, the items to be remembered were cubes. Changing the orientation of cubes (statically or dynamically) allows revealing its sides from different perspectives, which emphasizes their 3D structure and provides a vivid depth perception when these objects are presented in stereopsis as actual solids (instead of 2D reductions of the solids). Orientation also has previously been used as a feature to be detected in VSTM studies (Alvarez & Cavanagh, 2004; Harrison & Tong, 2009; Shin et al., 2017). Indeed, concerning VSTM for objects in the real world, one of the key factors in using 3D depth information is that this would seem particularly relevant for controlling our interactions with the remembered objects. For example, in reaching for objects out of sight (e.g., reaching for a tool behind or under something), information on their spatial orientation and 3D properties would seem particularly relevant.

Based on the previous studies and the above considerations, in the present study, we used the change-detection paradigm to measure VSTM and employed three different features (color, orientation, and direction-of-rotation) in current experiments. Visual stimuli were presented in stereopsis, by use of a VR set-up, where separate images were displayed to the two eyes to simulate stereoscopic vision in a 3D visual world. Our main goal was to explore the role of 3D depth information on VSTM in three conditions where we compare the VSTM performance: (i) monoscopic versus stereoscopic; (ii) conditions when objects share the same plane versus when objects are located in distinct depth planes; (iii) remembering an object’s non-spatial (color) feature (Experiment 1a) versus remembering its spatial features, like the orientation or direction-of-rotation of a cube (Experiments 1b and 1c).

## Experiment 1a

### Methods

#### Participants

Twenty-four individuals (12 female) volunteered to participate in the experiment. The sample size was determined according to power analyses computed by G*Power (Erdfelder et al., 2009) and the actual required number of participants was 18. The excepted effect size was 0.25 and alpha error was 0.05. One participant was excluded from the analyses due to recording failure. Mean age was 24.71 years (SD = 3.31 years). All participants had normal or corrected-to-normal vision with contact lenses. Informed consent from all participants before the experiment was collected. The participants of Experiment 1a also attended Experiment 1b and 1c.

#### Apparatus and stimuli

Images were presented using an HTC Vive HMD. The Vive has two OLED screens with separate 1,080 × 1,200-pixel resolution and a maximum 90-Hz refresh rate (fixed to 50 Hz in this study), and a 110-degree field of view. In this study, the refresh rate was fixed to 50 Hz. Participants responded by pressing the trigger buttons on an Xbox game controller.

All stimuli were generated and displayed using the *Unity* game engine (https://unity.com/). The memory stimuli were composed of a set of colored cubes with a 50-mm side-length. The number of cubes presented on a single trial ranged from three to five. Their colors were randomly selected from seven candidate colors without replacement. All cubes were presented in pseudorandom positions within a 4 × 6 grid subtending 24 × 36 degrees in the view field. In the single-plane condition, the distance between cubes and observer was 40 cm, while in the multi-plane condition, all items were located at separate distances from the observer, 30 cm, 34 cm, 38 cm, 42 cm, 46 cm, or 50 cm. No cubes were on the same plane.

Stereoscopic images are achieved in Unity by sending images from two separate virtual cameras to the two HMD screens. We emulated a monoscopic view by setting the distance between the two virtual cameras to zero. This resulted in identical images being rendered for each eye, and a “cyclopean” perspective onto the scene. The inter-pupil distance for each participant was set according to individual measurement prior to the experiment.

#### Procedure

The experiment started with a practice session, in which participants completed a set of examples (18 trials, about 3–4 min) to become familiar with the stimuli and procedure. In the main experiment, there were four blocks: monoscopic single-plane, stereoscopic single-plane, monoscopic multi-plane, and stereoscopic multi-plane. Each block contained 18 trials. For each participant, all blocks were arranged according to a Latin square design.

**Fig. 1:**
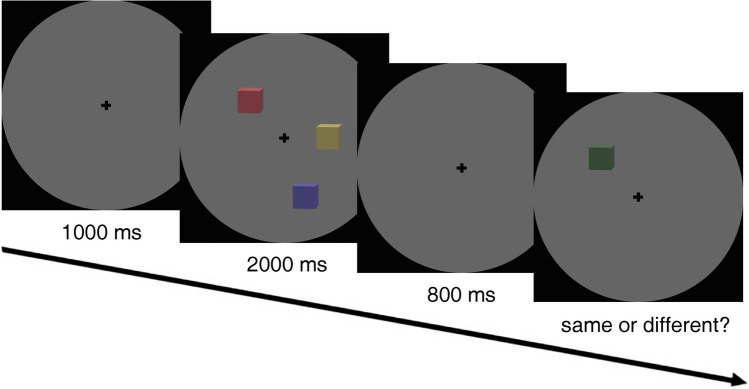
Illustration of the procedure for each trial in the “color feature” visual memory task. Participants judged whether the color of the cube that was re-presented in the same location remained the same or changed (in this example the correct answer would be “different”)

Each trial began with a gray fixation cross (1.15 visual degrees) being displayed for 1,000 ms and participants were instructed to keep their gaze on it. Subsequently, according to a pre-configured timeline, a memory array (three to five colored cubes) was presented for 2,000 ms. After an 800-ms blank screen, a single cube was presented on the screen until the observers responded. Participants were instructed to judge whether the color of the cube was identical to the cube that appeared in the same position in the memory array. Participants were asked to press the left trigger of an Xbox game controller if the color remained the “same,” and the opposite trigger if the color had changed or was “different.” Participants were also asked to respond as quickly and as accurately as possible. Between each trial, there was an interval lasting 2,000 ms (Fig. 1).

#### Statistics

The current study used a within-subject design, and the factors consisted of Stereo (monoscopic vs. stereoscopic), Depth Plane (single-plane vs. multi-plane) and Set Size (3 vs. 4 vs. 5). Standard repeated-measures ANOVAs were first performed. Subsequently, a Bayesian ANOVA approach (Bolstad, 2007; Kruschke, 2014; Marsman & Wagenmakers, 2017) was used to quantify the degree of support for the null hypothesis. Specifically, Bayes factors (BFs) are used as indicators of support for a given hypothesis. A BF_10_ larger than 3 can be interpreted as supporting H_1_ (or “alternate” hypothesis) while a BF_10_ smaller than 0.3 can be interpreted as supporting the “null” H_0_ (according to Dienes, 2014). Prior to statistical analyses, outliers defined by response time more than 1.5 interquartile ranges (IQRs) below the first quartile or above the third quartile were excluded (Tukey, 1977). In post hoc comparisons, Cohen’s *d* was calculated and used as a measure of effect size. The *p*-value was corrected for multiple testing by applying the Bonferroni correction.

### Results and discussion

**Fig. 2:**
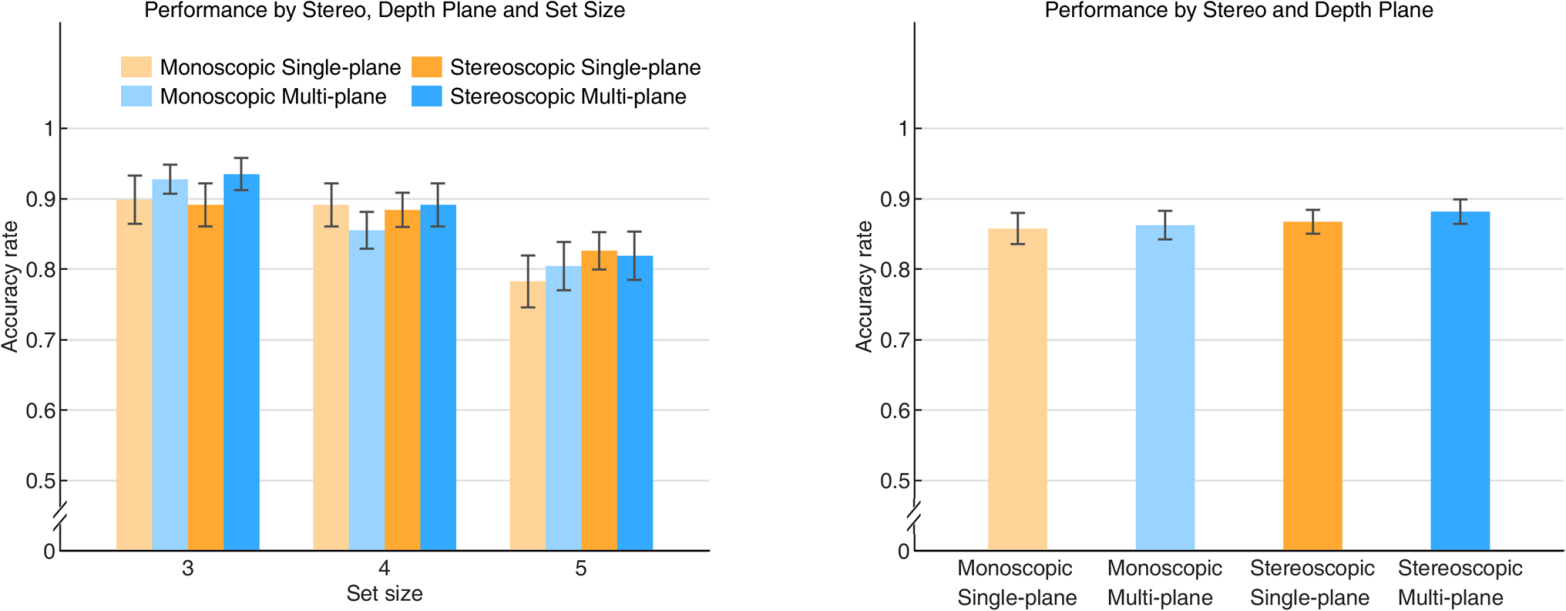
The accuracy rate for the color featured visual memory task. The left panel shows the results of the accuracy rate as a function of set size in all Stereo and Depth Plane conditions. In the right panel, the results of the accuracy rate were averaged across Set Size. Error bars indicate standard errors

The standard repeated-measures ANOVA and Bayesian repeated-measures ANOVA analyses revealed a main effect of Set Size, *F*(2, 44) = 10.196, *p* < 0.001, η^2^ = 0.317, BF_10_ > 1000. Not surprisingly, the accuracy rate decreased as the number of objects to be remembered increased. We did not find a significant interaction, in either type of analysis, between Set Size and Stereo, *F*(2, 44) = 0.455, *p* = 0.637, η^2^ = 0.020, BF_10_ = 0.095, or an interaction between Set size and Depth Plane, *F*(2, 44) = 1.466, *p* = 0.242, η^2^ = 0.062, BF_10_ = 0.178. There was no significant main effect of Stereo, *F*(1, 22) = 1.381, *p* = 0.252, η^2^ = 0.059, BF_10_ = 0.203, nor a main effect of Depth Plane, *F*(1, 22) = 0.593, *p* = 0.449, η^2^ = 0.026, BF_10_ = 0.158. The interaction between Stereo and Depth Plane was not significant either, *F*(1, 22) = 0.164, *p* = 0.689, η^2^ = 0.007, BF_10_ = 0.219. Furthermore, the three-factor interaction did not reach significance, *F*(2, 44) = 0.651, *p* = 0.527, η^2^ = 0.029, BF_10_ = 0.149 (see Fig. 2).

Experiment 1a showed that the accuracy rate did not significantly differ between the single-plane condition and the multi-plane condition. Note that the Bayesian analyses strengthened the conclusion in favor of the null hypothesis since a Bayes factor smaller than 0.3 (0.1 < BF_10_ < 0.3: moderately; BF_10_ < 0.1: strongly) is in favor of similarity among results (Bolstad, 2007; Dienes, 2014). The present findings are consistent with studies by Qian and Zhang (2019) and Reeves and Lei (2014). In Xu and Nakayama’s study (2007), the depth benefits in VSTM were only found when the color of objects and their spatial locations were correlated, that is, the effect of depth perception on VSTM was absent when there was no correlation. Qian and colleagues (2017) found the depth only improved VSTM for objects in the closer plane compared with objects in the more distant plane, and the two-plane condition did not improve VSTM performance over the single-plane condition. These two findings were both supported by our results that the multi-plane condition hardly contributes to the memory of colored objects independently. Another recent study found that the multi-plane condition improved the performance of VSTM when the size of memory array was larger than 4, suggesting that the benefit was possibly modulated by working memory load (Sarno et al., 2019). Combined with our findings in Experiment 1a, it appears that information about the 3D depth of an array of objects hardly contributes to the change detection accuracy for their color, or at least such a contribution is severely limited.

## Experiment 1b

Aiming to explore the effect of 3D depth on VSTM, we utilized the change-detection paradigm as determining whether the color of objects was changed or not in Experiment 1a but found no significant effect. We assumed that the color of objects is a less adequate feature to investigate the 3D depth’s benefits since color is a non-spatial feature. Thus, in the following experiment, we employed a spatial-relevant feature, the orientation of objects, and measured the VSTM performance in the Stereo and Depth Plane conditions.

### Methods

#### Stimuli and procedure

**Fig. 3:**
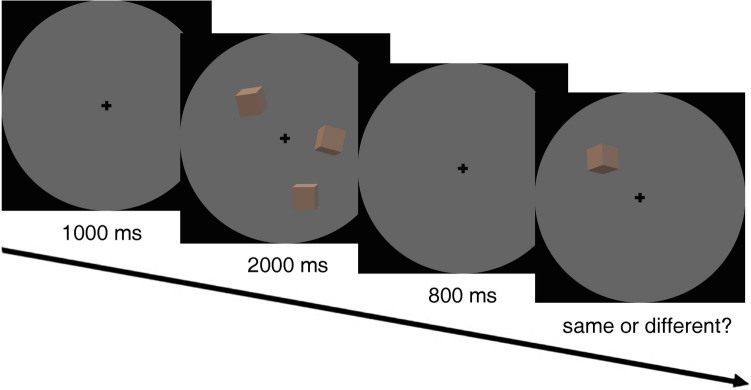
Illustration of the procedure for each trial in the orientation featured visual memory task. Participants judged whether the orientation of the cube that was re-presented at the same location remained the same or changed

The visual stimuli and experimental procedure in Experiment 1b were similar to Experiment 1a, for example, the size and number of cubes were identical to Experiment 1a, except for 4the following changes: The memory array was a set of cubes with the same color but different orientations; the directions in which the objects faced were randomly selected from six candidate orientations without replacement. Participants were instructed to judge whether the orientation of the cube was identical to the cube that appeared in the same position in the memory array. They pressed the left trigger if they judged the orientation remained the same, and the right trigger if they thought the orientation changed (Fig. 3).

### Results and discussion

In the repeated-measures ANOVA and repeated-measures Bayesian ANOVA analyses for accuracy rate, we found a main effect of Set Size, *F*(2, 44) = 9.860, *p* < 0.001, η^2^ = 0.309, BF_10_ = 486.352. This finding is consistent with previous work using color features; the increase of number of memory objects resulted in the decrease of the accuracy rate. There were no significant interaction between Set size and Stereo, *F*(2, 44) = 1.900, *p* = 0.162, η^2^ = 0.079, BF_10_ = 0.189, or interaction between Set size and Depth Plane, *F*(2, 44) = 0.531, *p* = 0.592, η^2^ = 0.024, BF_10_ = 0.118.

**Fig. 4:**
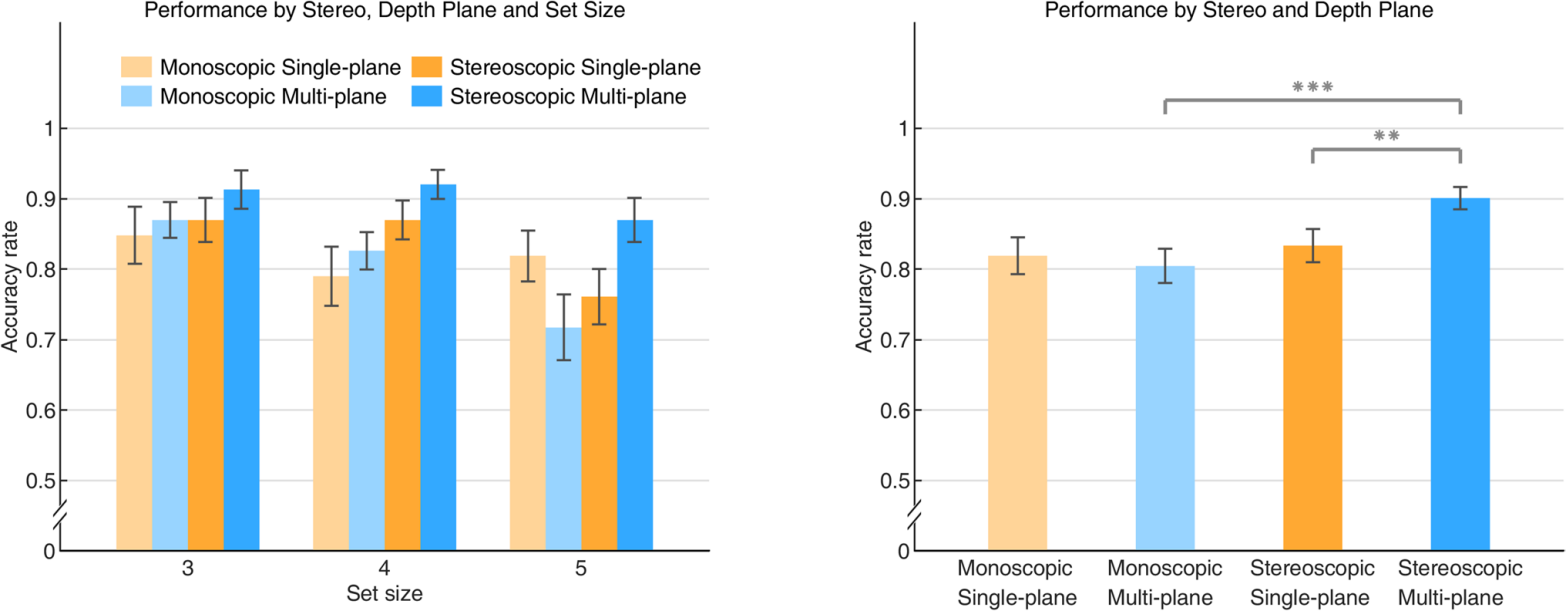
The accuracy rate for the orientation featured visual memory task. The left panel shows the results of the accuracy rate as a function of Set Size in all Stereo and Depth Plane conditions. In the right panel, the results of the accuracy rate were averaged across Set Size. Error bars indicate standard errors. *, *** indicate the corrected level of statistical significance of 0.05 and 0.001

Unlike our findings in Experiment 1a, the interactive effect between Stereo and Depth Plane was statistically significant, *F*(1, 22) = 10.457, *p* < 0.01, η^2^ = 0.322, BF_10_ = 4.264, revealing that the performance on VSTM in Depth Plane varied between the monoscopic and stereoscopic conditions. This finding can be accounted for by the significant difference in accuracy rate between the monoscopic multi-plane condition and stereoscopic multi-plane condition, mean difference = 0.097, SE = 0.017, *t*(22) = 5.741, *p*_*bonf*_ < 0.001, *d* = 1.197, and significant difference between accuracy rate in the stereoscopic single-plane and stereoscopic multi-plane conditions, mean difference = 0.068, SE = 0.023, *t*(22) = 2.921, *p*_*bonf*_ = 0.016, *d* = 0.652. This result showed a superior performance in the stereoscopic multi-plane condition and provided evidence that VSTM performance could benefit from 3D depth when presented in stereopsis. However, the difference between accuracy rate in the monoscopic single-plane condition and monoscopic multi-plane condition was not significant, mean difference = 0.014, SE = 0.021, *t*(22) = 0.680, *p*_*bonf*_ = 1, *d* = 0.142, which indicated that a difference in relative size of objects did not necessarily result in a difference of VSTM performance (see Fig. 4).

Additionally, the main effect of Stereo was significant, *F*(1, 22) = 11.768, *p* < 0.01, η^2^ = 0.348, BF_10_ = 47.381. Post hoc comparisons showed that the accuracy rate in the stereoscopic condition was significantly larger than that in the monoscopic condition, mean difference = 0.056, SE = 0.016, *t*(22) = 3.430, *p*_*bonf*_ = 0.002, *d* = 0.715. However, The main effect of Depth Plane did not reach significance, *F*(1, 22) = 2.424, *p* = 0.134, η^2^ = 0.099, BF_10_ = 0.524. No significant difference was found between single-plane and multi-plane, mean difference = 0.027, SE = 0.017, *t*(22) = 1.557, *p*_*bonf*_ = 0.134, *d* = 0.325. We also found a significant three-way interaction across all factors, *F*(2, 44) = 5.492, *p* < 0.01, η^2^ = 0.200, BF_10_ = 4.932, which appeared to be due to an unexpected increase from size 4 to 5 in the monoscopic single-plane condition.

## Experiment 1c

The results of Experiment 1b showed that distance depth planes improved VSTM performance in stereopsis when orientation was the feature to be remembered in a change-detection paradigm. In order to further test the effects of 3D depth on VSTM, we made the cubes dynamically rotate around one of the X, Y, or Z axes and instructed participants to detect these changes in direction-of-rotation.

### Methods

#### Stimuli and procedure

**Fig. 5:**
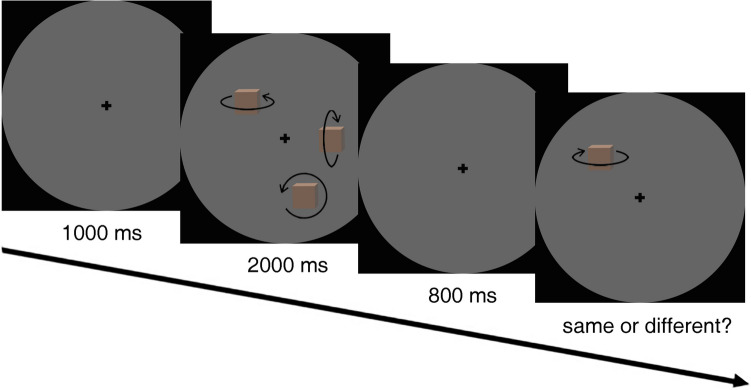
Illustration of the procedure for each trial in the direction-of-rotation featured visual memory task. Participants judged whether the direction-of-rotation of the cube when re-presented at the same location remained the same or changed. The arrows in the figure are only for illustrating here the direction of rotations and were not displayed in the experiment

The stimuli and experimental procedures were similar to Experiments 1a and 1b, i.e., the memory array was a set of cubes with the same color, except for the following changes. Each cube was actively rotating around one of the X, Y, or Z axes at the speed of 90°/s; the size and number of cubes were identical to Experiments 1a and 1b. Participants were instructed to judge whether the direction-of-rotation of the cube was identical to the direction it rotated when the cube appeared in the same position in the memory array. The direction-of-rotation can only change from clockwise to anticlockwise or from anticlockwise to clockwise, and never change the axis of rotation (Fig. 5).

### Results and discussion

In both a repeated-measures ANOVA and a repeated-measures Bayesian ANOVA for accuracy rate, we found a strong main effect of Set Size, *F*(2, 44) = 39.286, *p* < 0.001, η^2^ = 0.641, BF_10_ > 1000. There was no significant interaction between Set Size and Stereo, *F*(2, 44) = 1.993, *p* = 0.148, η^2^ = 0.083, BF_10_ = 0.305, or interaction between Set size and Depth Plane, *F*(2, 44) = 0.531, *p* = 0.592, η^2^ = 0.024, BF_10_ = 0.104. Consistent with the results of Experiment 1b, we found a significant main effect of Stereo, *F*(1, 22) = 9.274, *p* < 0.01, η^2^ = 0.297, BF_10_ = 3.157, and a non-significant main effect of Depth Plane, *F*(1, 22) = 3.364, *p* = 0.080, η^2^ = 0.133, BF_10_ = 0.628.

**Fig. 6:**
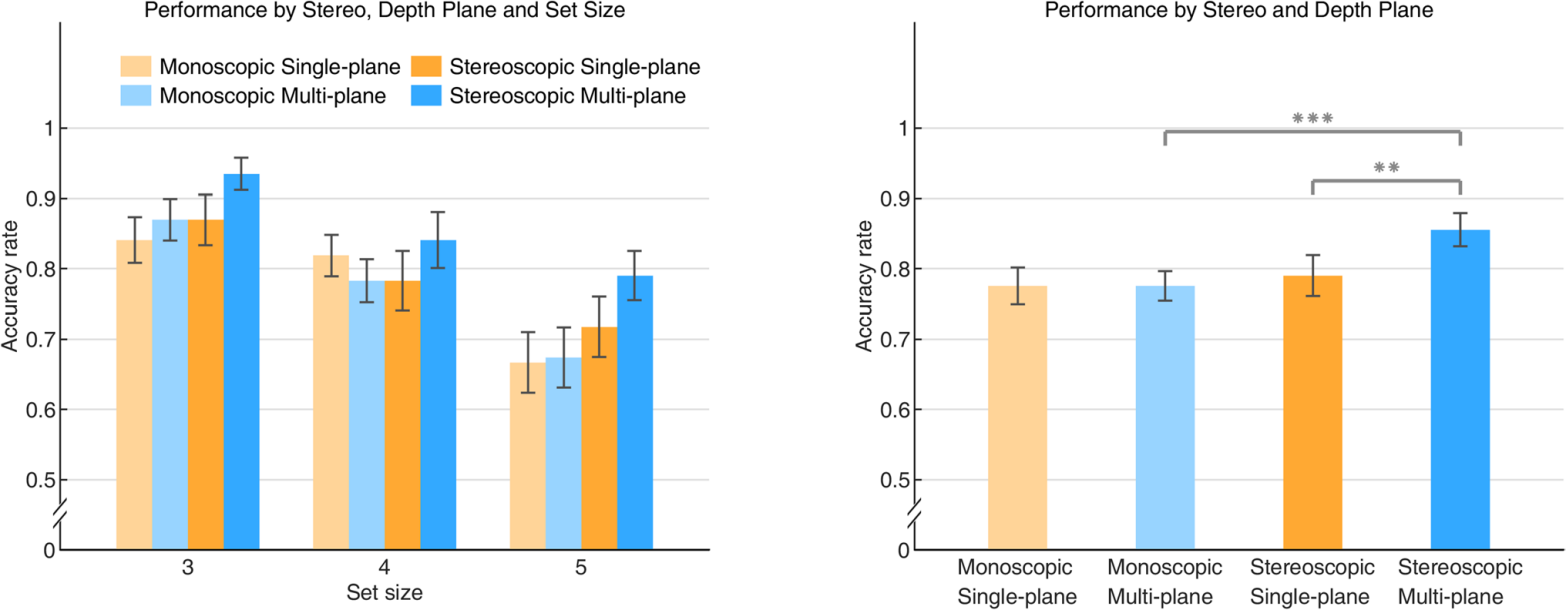
The accuracy rate for the direction-of-rotation featured visual memory task. The left panel shows the results of the accuracy rate as a function of set size in all Stereo and Depth Plane conditions. In the right panel, the results of the accuracy rate were averaged across Set Size. Error bars indicate standard errors. **, *** indicate the corrected level of statistical significance of 0.01 and 0.001

However, there was no significant interaction between Stereo and Depth Plane, *F*(1, 22) = 3.804, *p* = 0.064, η^2^ = 0.147, BF_10_ = 0.958, but interestingly, we found similar patterns in the VSTM performance for Experiments 1b and 1c, where the change detection accuracy for orientation and direction-of-rotation were significantly superior in the stereoscopic multi-plane condition. This was accounted for by the results showing that the accuracy rate in the stereoscopic multi-plane condition was significantly larger than that in the monoscopic multi-plane condition, mean difference = 0.080, SE = 0.017, *t*(22) = 4.580, *p*_*bonf*_ < 0.001, *d* = 0.955, and also larger than that in the stereoscopic single-plane condition, mean difference = 0.065, SE = 0.021, *t*(22) = 3.127, *p*_*bonf*_ = 0.010, *d* = 0.652. A potential explanation may be that there is only an advantage of 3D depth when spatial perception is required for completing the tasks. Both presenting cubes from various perspectives (Experiment 1b) and making them rotate around one of the axes (Experiment 1c) emphasizes their 3D structure. When detecting such features, orientation and direction-of-rotation, all participants coded the depth structure of objects per se and the spatial information of objects in depth (only available in stereoscopic multi-plane condition) together, which made the benefits of depth available. Finally, as expected, the three-way interaction between all factors did not reach significance, *F*(2, 44) = 0.260, *p* = 0.773, η^2^ = 0.012, BF_10_ = 0.139 (see Fig. 6).

## General discussion

The present study used a VR display and a change-detection paradigm to investigate the influence of 3D depth perception or stereopsis on VSTM performance. In three experiments, participants were requested to detect changes in color, orientation, or direction-of-rotation of cubes, which were presented either in the same vertical plane in space or in individually differing depth planes. We reasoned that remembering a non-spatial feature like color would not benefit from the presence of spatial information of depth and performance would be similar regardless of whether the stimuli were in a spatially deep array or not. Indeed, in Experiment 1a, where color was the relevant feature, we found that there were no benefits to seeing the scene in depth, since we could not detect a difference between the single and multiplane conditions and Bayesian analyses confirmed a performance similarity. However, in Experiments 1b and 1c, where we used two spatially relevant features, we found that the distinct depth planes in stereopsis clearly facilitated VSTM. As expected, we found that set size (i.e., number of cubes) also had an impact on memory, and that the accuracy rate decreased as the number of objects in memory array increased.

Previous studies have shown that displaying stimuli in stereopsis, or in visual ecologically valid conditions, improved performance on a variety of visual tasks (e.g., Chelnokova & Laeng, 2011; Finlayson & Grove, 2015; Lim Lee & Saunders, 2011; Umemura, 2015). These researchers suggested that the observed perceptual benefits were due to the specific visual information added by binocular disparity (Finlayson & Golomb, 2016). Inspired by these previous studies, we directly compared the performance on VSTM in the stereoscopic single-plane and monoscopic single-plane condition. We confirmed, also with Bayesian analyses, that stereoscopic information hardly contributes to VSTM when objects in an array are viewed on the same single plane. Note that this is how the great majority of visual experiments in perception present stimuli, appearing on a same vertical plane in space, despite this being only one of the possible arrangements in ecological viewing conditions, and probably not the most common in our daily experience.

In contrast, in both Experiment 1b and Experiment 1c, the change detection accuracy for orientation and direction-of-rotation were significantly superior in the stereoscopic multi-plane condition compared to the monoscopic multi-plane condition. In other words, when the change occurred for two spatial features, we found similar advantages in VSTM performance with depth information. Some researchers have suggested that the 3D depth could improve behavioral performance by providing some sort of group cueing or “memoranda” (Chunharas et al., 2019; Qian et al., 2017; Xu & Nakayama, 2007). Thus, participants would be able to maintain more items in mind because grouping them would reduce the processing load on memory (Peterson & Berryhill, 2013). However, one should note that in the present study we had set as a contrast a “monoscopic” condition where the images for two eyes were identical. Thus, in the monoscopic multi-plane condition, although objects could appear as located in distinct depth planes, observers were unable to perceive their depth directly due to the absence of binocular disparity and therefore “inferred” from the relative sizes of objects (i.e., a monocular depth cue). Since we found that the performance in the “relative size” condition was not superior compared to the single-plane condition, the present results are in accordance with previous work by Qian and colleagues’ study (2017), who found that only the cooperation between size grouping and disparity depth cue does improve performance. Taken together, the present findings support the conclusion that depth benefits may not be due to the grouping effects on arrays of relatively different size, but that the benefits on memory are likely associated with the added 3D depth information provided by stereopsis.

Moreover, there is independent evidence demonstrating that the presence of distinct depth planes improves performance in other visual tasks. For example, visual search studies have indicated that separation in depth improved the performance on visual search tasks (Dent et al., 2012; Finlayson & Grove, 2015; Godwin et al., 2017). The benefit of depth on VSTM was observed in both low-capacity and high-capacity groups, indicating that depth information is generally helpful across individuals (Sarno et al., 2019). In light of those results and our findings in Experiments 1b and 1c, we suggest that the perceptual benefits of depth in these various visual tasks may arise from a common mechanism that is based on depth benefits accrued by the presence of spatial relevant features. In particular, when the feature of objects to be remembered is spatially relevant, coding such spatial features in 3D and in depth space can facilitate VSTM.

In fact, we did not find benefits from stereoscopic depth in Experiment 1a, where processing the objects’ color feature was essential but depth information was apparently irrelevant for performance. Thus, color may not be an optimal feature for exploring the role of depth perception on VSTM, and it may not be surprising that previous studies yielded mixed findings (Chunharas et al., 2019; Qian et al., 2017, 2018; Sarno et al., 2019; Xu & Nakayama, 2007). Note that the occasional findings of depth effect, as in Chunharas and colleagues’ study (2019), could be due to the fact that although they used the color of objects as a change detection feature, they also utilized a depth-discrimination task where observers could code the spatial information rapidly, and perhaps not surprisingly performance on VSTM benefited from 3D depth.

To conclude, performance on VSTM is modulated by 3D depth when the relevant feature is spatial. In Experiment 1a, we employed the color feature in a change-detection task and found no difference between the single-plane condition and the multi-plane condition either in the monoscopic condition or in the stereoscopic condition. The effect of depth perception on VSTM was severely limited, likely because the color of objects is a non-spatial feature. In Experiments 1b and 1c, we conducted similar tasks but replaced the color feature in the change-detection task with orientation and direction-of-rotation of objects. We found the accuracy rate to be significantly higher in the stereoscopic multi-plane condition, indicating that the performance on VSTM benefits from the presence of 3D depth information. To sum up, our findings suggest that the 3D depth in stereopsis confers an important benefit to VSTM when spatial perception is required, while the contribution is minor when the relevant feature for the task is non-spatial.

## Data Availability

The data for the experiments reported here are available, and the experimental materials can be available when required. None of the experiments was preregistered. Identifier: DOI 10.17605/OSF.IO/JFZHK.

## Code availability

The data are currently available from the first author and will be placed in a repository when required.
